# Modeling of residual chlorine in a drinking water network in times of pandemic of the SARS-CoV-2 (COVID-19)

**DOI:** 10.1186/s42834-021-00084-w

**Published:** 2021-03-11

**Authors:** Fernando García-Ávila, Alex Avilés-Añazco, Juan Ordoñez-Jara, Christian Guanuchi-Quezada, Lisveth Flores del Pino, Lía Ramos-Fernández

**Affiliations:** 1 Faculty of Chemical Sciences, University of Cuenca, 010107 Cuenca, Ecuador; 2 Science Faculty, La Molina Agrarian National University, 15023 Lima, Peru; 3 Department of Water Resources, La Molina Agrarian National University, 15023 Lima, Peru

**Keywords:** Bulk decay constant, COVID-19, Disinfectant, Residual chlorine, Wall decay rate

## Abstract

Due to the outbreak of the novel coronavirus disease there is a need for public water supply of the highest quality. Adequate levels of chlorine allow immediate elimination of harmful bacteria and viruses and provide a protective residual throughout the drinking water distribution network (DWDN). Therefore, a residual chlorine decay model was developed to predict chlorine levels in a real drinking water distribution network. The model allowed determining human exposure to drinking water with a deficit of residual chlorine, considering that it is currently necessary for the population to have clean water to combat coronavirus Covid 19. The chlorine bulk decay rates (kb) and the reaction constant of chlorine with the pipe wall (kw) were experimentally determined. Average kb and kw values of 3.7 d^− 1^ and 0.066 m d^− 1^ were obtained, respectively. The values of kb and kw were used in EPANET to simulate the chlorine concentrations in a DWDN. The residual chlorine concentrations simulated by the properly calibrated and validated model were notably close to the actual concentrations measured at different points of the DWDN. The results showed that maintaining a chlorine concentration of 0.87 mg L^− 1^ in the distribution tank, the residual chlorine values in the nodes complied with the Ecuadorian standard (0.3 mg L^− 1^); meanwhile, about 45% of the nodes did not comply with what is recommended by the WHO as a mechanism to combat the current pandemic (0.5 mg L^− 1^). This study demonstrated that residual chlorine modeling is a valuable tool for monitoring water quality in the distribution network, allowing to control residual chlorine levels in this pandemic season.

## Introduction

Providing quality drinking water is a critical component in response to a sanitary emergency, and chlorination is widely used in emergencies to treat water [1]. In a drinking water distribution network (DWDN), disinfection with chlorine is important to prevent the spread of waterborne diseases as a result of bacteria and viruses [2, 3]. Waterborne viral pathogens are classified by the World Health Organization (WHO) as being of moderate to high importance to health, including adenoviruses, astroviruses, hepatitis A and E viruses, rotaviruses, noroviruses, and others. In the past months, another virus has emerged, the novel coronavirus (SARS-CoV-2), which causes the disease called COVID-19. It is indicated that this virus cannot be transmitted through drinking water, however, the evidence is not conclusive [3, 4]. Currently, the availability of clean water is necessary to face the health situation that the world is going through due to SARS-CoV-2. Washing hands, showering, cleaning and disinfecting houses requires good quality water [5]. Therefore, the presence of residual chlorine of 0.5 mg L^− 1^, measured at the endpoints of the water distribution system, must be guaranteed [5, 6]. A valuable tool to ensure residual chlorine levels in the DWDN is to develop a model to predict the level of disinfectant.

The water quality can deteriorate in the distribution system after the water leaves the treatment plant [7]. While the water flows through pipelines, the chlorine concentration decays because the chlorine reacts with organic and inorganic compounds present in the water and with the pipeline wall [8, 9]. If the concentration of residual chlorine in the water is too low, it may not provide effective protection against recontamination. Too high a chlorine level can lead to consumer complaints and pipe network corrosion. On the other hand, even a lower amount of Cl_2_ causes the formation of disinfection by-products [10, 11]. Reactions that lead to the decrease in chlorine concentration occur in the liquid phase and at the liquid-solid interface between the water and the internal walls of the pipeline [12, 13].

Free chlorine is the most non-conservative substance used in water quality models and is modelled with a first-order chlorine decay reaction, assuming that chlorine concentration decreases exponentially [14].

To model the decay of chlorine in a distribution network, the reaction rate of chlorine with the mass of water (kb, bulk decay) must be considered; in this case, there are chemical reactions of chlorine with the natural organic matter present in the water [15, 16]. Likewise, it must be considered that the substances contained in the water that circulates in a pipeline can be transported to the wall of the pipeline and react with the chlorine [17–19]. The reaction of chlorine with the pipeline wall (kw) is normally measured in terms of reaction rate and depends on the amount of surface available to react and the mass transfer rate between the fluid and the pipeline wall [15, 20, 21]. Hallam discovered that the nature of the pipeline material has a strong effect on the chlorine problem during water distribution [17]. Previous studies reported that synthetic materials, such as PVC, have a very low chlorine demand [18].

In times of Covid 19 pandemic, the implementation of residual chlorine decay models in distribution networks is necessary to ensure the drinking water quality. By implementing these models, the cost of spatial and temporal monitoring of the residual chlorine in the supply systems can be reduced [22, 23]. With the implementation of a residual chlorine model, control of the disinfectant level could be improved, which is essential to combat diseases such as the current COVID-19 pandemic or cholera [24, 25]. Likewise, it would prevent the technical staff of the supplier companies from having to travel to the different points of the network to carry out the monitoring, which would help maintain social distancing in times of the COVID-19 pandemic. Likewise, it will allow monitoring of chlorine levels at all points in the network and identify the human population that is possibly exposed to waters with residual chlorine levels below the standard.

US Environmental Protection Agency (EPA) [26] indicates the need for filtration and disinfection with chlorine, to eliminate waterborne pathogens, such as viruses. SARS-CoV-2, being an enveloped virus, does not easily survive in water, and can be removed and inactivated by contact with chlorine [27].

The health emergency caused by COVID-19 has shown the importance of disinfection in the treatment of drinking water, so it is necessary to maintain the optimal dose of residual chlorine in the DWDN to protect public health. There is no evidence to date on the survival of the virus in drinking water, it is likely that the virus in contact with chlorine is inactivated significantly faster than enteric viruses [28].

Modelling of chlorine decay is still complex and requires a good understanding of the system together with a properly calibrated and validated hydraulic model [18] and accurate values of the kinetic constants required for the decay model of residual chlorine [19].

The objective of the present work was to develop a residual chlorine decay model that allows controlling the disinfectant levels in a real distribution network using EPANET [29]. EPANET is a software developed by the US EPA that allows simulations of hydraulic behavior and the evolution of water quality in pressure supply networks.

The developed model allowed the simulation of residual chlorine concentrations in the DWDN of the city of Azogues in Ecuador. The results of the simulation made it possible to establish whether the WHO recommended residual chlorine level is currently being met as a mechanism to combat COVID-19. In addition, the results of this study can be used in other drinking water distribution networks, in order to guarantee the availability of clean water, which has now become a fundamental element to combat the novel coronavirus, for washing hands, showering, as well as cleaning and disinfecting homes.

## Materials and methods

### Study area

The residual chlorine decay constants were determined experimentally in the distribution network of Azogues city, Ecuador. The city has an irregular topography with an average elevation of 2500 m above sea level. The distributed drinking water is obtained after a process consisting of coagulation/flocculation/sedimentation, sand-anthracite filtration and final disinfection with gas chlorine. The average chlorine content in the distribution tank is 0.87 mg L^− 1^. The physicochemical characteristics of the water distributed in this network have been reported in the study carried out by García-Avila et al. [30]. The average values reported are: Turbidity = 0.51 NTU, pH = 7.24, Electrical conductivity = 110.47 μS cm^− 1^, Total hardness = 69 mg L^− 1^ as CaCO_3_, Alkalinity = 48 mg L^− 1^ as CaCO_3_, Sulfates = 18 mg L^− 1^, Nitrates = 0.48 mg L^− 1^, Phosphates = 0.08 mg L^− 1^, Chlorides = 5.6 mg L^− 1^.

**Fig. 1:**
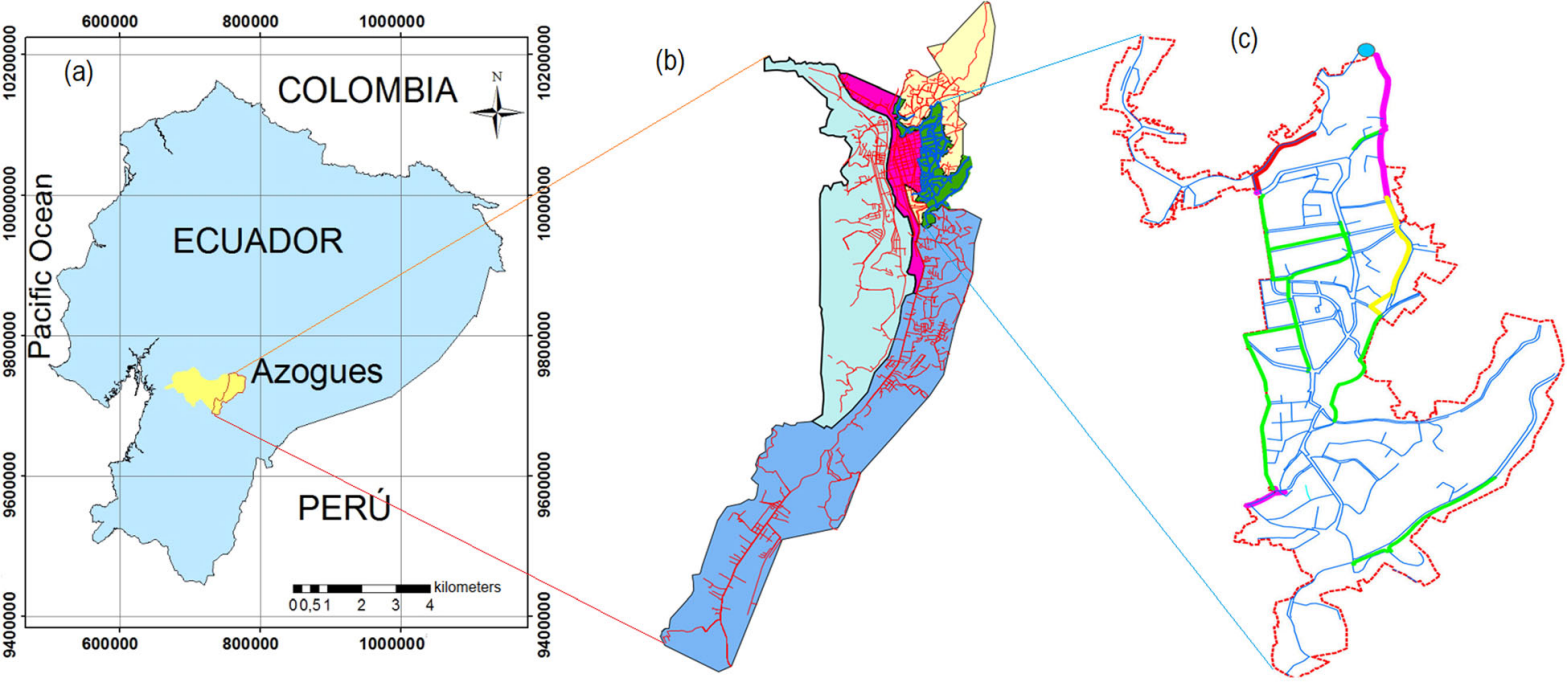
Location of the study area. **a** Location of the Azogues city, Republic of Ecuador. The coordinate reference system is WGS84/UTM zone 17S EPSG 32717 **b** Total drinking water network in the city, **c** Drinking water distribution network chosen for the study

The modeling was performed in the upper zone of the distribution network of the city of Azogues. The network is the open type, and the supply is continuous, for which it has a reservoir of 500 m^3^, and the distribution is carried out by gravity. The system consists of PVC pipes, with the diameters ranging from 32 to 315 mm. The system has 380 nodes and 370 pipelines with a total network length of 26.6 km (Fig. 1). The average age of this PVC pipeline network is approximately 10 yr.

### Hydraulic modelling

Proper hydraulic modelling is a prerequisite for modelling of water quality [31]. For the present study, the hydraulic model developed in EPANET by García-Avila et al. [7] was used. This hydraulic model was duly calibrated and validated for this same study place and applied as a tool to reduce leaks [7]. Furthermore, this hydraulic model had: (1) information on the distribution network to be modeled, in terms of diameters, pipe lengths and pipe materials; (2) demand patterns for each node in the system; and (3) an extension of hydraulic software to implement the equations of the chlorine decay model.

In this hydraulic model, the evolutions of the flows and speeds in the pipes, the pressures in the nodes, the levels in the tank were previously calculated.

### Formulation of the chlorine decay model

For the bulk chlorine decay modeling, a first-order reaction kinetics was considered [21, 29]. For the reaction of chlorine with the wall in non-metallic pipes, a first-order kinetic model was used [15, 16, 21]. It was also necessary to establish the value of the relative diffusivity, which was set as 1, because this value is defined as a function of the relative diffusivity with respect to chlorine [29]. A chlorine concentration pattern was developed at the outlet of the distribution tank, 24 h a day. For the calibration and validation of the model, field measurements of chlorine were carried out at various points of the distribution network.

Therefore, to model the residual chlorine decay with EPANET, predetermined kb and kw values are of essential [16, 22].

#### Bulk decay coefficient (kb)

There is no standard test to measure the chlorine bulk decay coefficient, kb. To determine this coefficient, an experimental procedure called bottle testing was used [9, 32]. For this procedure, once the sample was collected, the chlorine concentration was measured, and the hour of onset were recorded (measurement in the field). Subsequently, the chlorine concentration was measured at intervals of 1 h until the chlorine concentration tended to zero (measurement in the laboratory). With this test, kb was determined due to the reaction of chlorine with the mass of water (chemical reactions of chlorine with natural organic matter present in water).

The measured data were processed by curve fitting using Excel, and an exponential type decay curve was constructed [21, 33]. We determined kb after the exponential curve fitting, using the equation: $\mathrm{C}={\mathrm{C}}_0{\mathrm{e}}^{-{\mathrm{k}}_{\mathrm{b}}\mathrm{t}}$. For chlorine determination, a portable digital measuring device (DR 890 HACH) was used based on the DPD colorimetric determination [16, 34].

Water sampling points used to evaluate kb were determined strategically after the network plans were revised, and field trips were made in order to determine the most suitable testing locations; the length of the distribution network, the locations of the reservoirs and the number of consumers that comprise the supply line were taken into account. The sampling points in this study were: household taps; commercial premises such as restaurants, workshops, car washers, shops, etc., as well as in the distribution tanks.

Thirty sampling points were selected based on the abovementioned considerations. Reservoirs, homes, commercial premises, and educational units were chosen. Sampling plans were prepared to collect the 30 monthly samples for 6 months. A total of 180 samples were collected in 1000 mL plastic bottles so as not to alter the samples for further analysis. The bottles were prepared previously as recommended by the Ecuadorian standard [35]. The containers were washed with a calcium hypochlorite concentrated solution with the concentration of 10 mg L^− 1^ and were left to stand for 24 h. Then, the containers were emptied, rinsed thoroughly with distilled water and allowed to dry.

Water samples were taken from the tap of the consumers who were directly connected to the distribution network, and the water was allowed to run for approximately 2 min in order to avoid collecting water that had been unused for a long period of time. The bottles were stored in an incubator in order to be stored at a constant temperature. All of the sampling and preservation procedures were carried out according to the standard methods for the water and wastewater examination of APHA [36]. The sampling campaigns were carried out in the months of July, August and September, which are the months with the lowest temperature and during the months of January, February and March, which are the months with the highest temperature.

#### Wall decay coefficient (kw)

The kw depends on the actual environment characteristics of the pipeline such as the material type, diameter, roughness, age, biofilm formation and water temperature, which makes it difficult to measure the decay coefficient in the laboratory. To calculate kw, Eq. (1) was used, for which it was necessary to previously known kb, kf and K [15, 37].

1 $$ K={k}_b+\frac{2{k}_w{k}_f}{R_h\left({k}_w+{k}_f\right)} $$

where, K is the total decay constant (d^− 1^), kw is the wall reaction rate constant (m d^− 1^), kf is the mass transfer coefficient (m d^− 1^), and Rh is the hydraulic radius of the pipe (m). In section 2.3.1 it was already indicated how to determine kb. K was determined experimentally, as indicated in the following section.

The kf etween the water flow and the wall was calculated using Eq. (2) [15]:

2 $$ {k}_f=\frac{S_hD}{d} $$

where, Sh is the Sherwood number, D is the molecular diffusivity of the substance within the fluid, and d is the pipeline diameter. To determine the Sherwood number, the recommendations of Vasconcelos et al. [15] were followed.

#### Total decay constant K

The K coefficient was determined by field tests for which primary network pipes (without branches) of constant diameter and known length were selected [34]. The chlorine concentration was measured at the ends of each pipe, and the flow rate that was used to calculate the speed was also determined. The first-order general decay rate constant was calculated according to Eq. (3) [17]:

3 $$ \mathrm{K}=\frac{v}{L}\ln \frac{C}{C_0} $$

here, L is the length of the pipeline section, m, v is the velocity of the flow within the pipeline stretch, m s^− 1^, C is the concentration of the substance at the end of the pipeline stretch, mg L^− 1^, and C_0_ is the concentration of the substance at the beginning of the stretch, mg L^− 1^.

Six pipes with different diameters (250, 200, 200, 160, 110 and 63 mm) were used for the field tests, of which, two were of the same diameter (200 mm) with the lengths of 3075, 416, 3380, 1817, 2535 and 2510 m, respectively. These pipes carry water from the flow distributor (single source) to the distribution tanks and the path of the water distribution does not have ramifications. The tests were performed once a day for 5 weeks. The results were averaged to obtain the K for each pipe.

### Model calibration and validation

The experimentally obtained average values of kb and kw were initially entered into EPANET. The model was calibrated by adjusting the values of kb and/or kw in some pipes of the network until the best fit was obtained between the measurements of the chlorine obtained in the field and the simulated values. In some pipes, the kb values were modified and were close to the average value initially entered. Likewise, kw was adjusted as the flow velocities obtained in the hydraulic model were previously analyzed and the kw values were considered in correspondence to their respective velocities for each pipeline. To evaluate the efficiency of the calibration and validation, the Nash-Sutcliffe index (E), the standardized mean square error (RSR) and the Pearson correlation coefficient (R) defined by [38] were used. For the calibration of the model, 20 chlorine measurements were used. After calibration, the model was validated. For this purpose, a new chlorine measurement programme was carried out at 11 points in the supply network. Once the model was validated, water quality analysis was carried out for a prolonged period based on the chlorine component [39].

### Sensitivity analysis of the chlorine decay model

The sensitivity analysis of the chlorine decay model was carried out using the traditional method of parameters perturbation, that is, a parameter of the model was varied while the rest of the parameters remain constant, so that the variations observed in the state variables reflect the sensitivity of the solution to the modified parameter [40]. A sensitivity analysis was carried out following the technique described by Chen et al. [41] and Wang et al. [42].

## Results and discussion

### Bulk decay kinetic coefficient

**Fig. 2:**
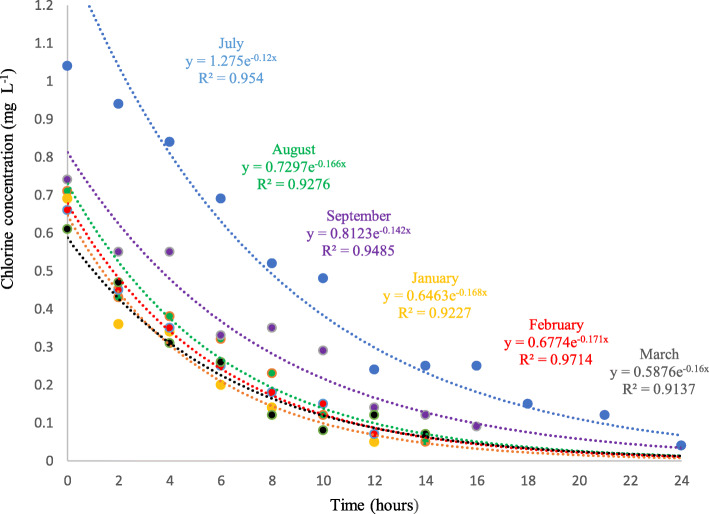
Chlorine decomposition coefficients in mass at different initial chlorine concentrations. An exponential relationship was obtained between the chlorine concentration (C) and the elapsed time (t) in each sample collected during the 6 months

The experimental results and the best fit to determine kb are presented in Fig. 2, and it is observed that starting from an initial concentration C_0_, the residual chlorine decays with time (t). An exponential relationship was obtained between the chlorine concentration (C) and the elapsed time (t) for each initial chlorine concentration (C_0_). kb was estimated in the 180 samples collected during the 6 months. As an example, the relationship between C_0_ and t of a sampling point for each month is presented in Fig. 2. Following this procedure, the kb values were determined for all of the samples of the 6 months monitored. The negative sign in the equation refers to the decrease in the residual chlorine over time. The results of the chlorine mass decomposition coefficient experiments given in Fig. 2 support the assumption of the first-order chlorine disintegration kinetics. Table 1 presents the monthly average of the kb and it is noted that kb was lower (0.12 h^− 1^) in July (minimum temperature), while the highest coefficient with a value of 0.19 h^− 1^ was observed in March (maximum temperature). Chlorine decay constants are temperature dependent: the higher the temperature, the higher the decay rate constant.

**Table 1:** Average monthly coefficient kb

Month	kb (h^−1^)	kb (d^− 1^)	T (°C)
July	0.12	3.0	15.7
August	0.13	3.1	16.3
September	0.16	3.9	17.1
January	0.13	3.7	18.7
February	0.16	3.9	18.8
March	0.19	4.6	19.4
Average	0.15	3.7	17.7

An average value of the constant kb = 0.15 h^− 1^ was obtained for the study area. This value is higher than other studies (Table 2). The latest results presented in Table 2 are similar to those obtained in this study and indicate the presence of different concentrations of total organic carbon in the analyzed samples. The variation of kb is observed because the reaction depends on the particular conditions of each zone.

**Table 2:** kb values obtained by different authors. In this study, a kb = 0.15 h^−1^ was obtained, which is higher than most of the values reported in this table

Authors	kb
Rossman et al. [16]	0.55 d^− 1^ (0.0229 h^− 1^)
Fisher et al. [21]	0.27 d^− 1^ (0.011 h^− 1^)
Rossman and Boulos [43]	2.0 d^− 1^ (0.083 h^− 1^)
Hua et al. [44]	0.02–0.09 h^− 1^
Abokifa et al. [45]	0.55 d^− 1^ (0.022 h^− 1^)
Grayman et al. [22]	0.5 d^− 1^ (0.020 h^− 1^) for river water
	5.0 d^−1^ (0.20 h^− 1^) for lake water
Diagiano and Zhang [46]	0.033 h^− 1^
Araya and Sanchez [47]	0.05 d^− 1^ (0.002 h^− 1^)
Mostafa et al. [48]	0.033 h^− 1^
Alcocer-Yamanaka et al. [49]	0.098 h^− 1^
Ammar et al. [50]	1.72–2.07 d^− 1^ (0.071–0.086 h^− 1^)
Vasconcelos et al. [15]	0.082–17.7 d^− 1^ (0.034–0.74 h^− 1^)
Courtis et al. [51]	0.033–0.226 h^− 1^

### Total decay coefficient K

All of the tests were performed on PVC pipes that were in operation for 10 yr approximately. The 110 mm diameter pipeline is the sole exception to this rule is where a newly installed pipeline was used (1 month of operation). The final global decay coefficient was obtained by finding the average of the values obtained in each test in the different diameter pipelines. The experimental results for K are shown in Table 3.

**Table 3:** Experimental results obtained when determining the total decay coefficient K

Diameter (mm)	Flow (m^3^ s^− 1^)	Velocity (m s^− 1^)	Length (m)	K (h^− 1^)
250	0.0725	1.43	3075	0.226
200	0.0352	1.09	416	0.222
200	0.0219	0.675	3380	0.219
160	0.0304	1.51	1817	0.271
110	0.0003	0.10	2535	0.157
63	0.0009	0.11	2510	0.159

A multiple linear regression analysis to analyze the relationship between K with diameter, flow, velocity and length was performed. Velocity and flow were determined to have *p* < 0.05, that is, they have a significant linear relationship with K. The diameter and length showed *p* > 0.05, that is, their linear relationship with K was not significant. Once these last two variables were eliminated, a new linear regression analysis was performed; verifying that K depends mainly on the speed of the water and to a lesser extent depends on the flow. The empirical formula *K = 0.16 + 0.06u* was obtained, where u is the flow velocity in m s^− 1^. According to the obtained results, the chlorine decay rates with the wall depend on the flow velocities and on the pipes diameter. However, the total decrease in chlorine concentration has a stronger relationship with the flow velocity.

### Wall decay coefficient kw

The coefficient kw was determined using Eq. (1). The results are presented in Table 4. From the coefficients kb and kw obtained for all the pipes under study, it can be inferred that the chlorine decay in this study was predominantly due to the reactions of the disinfectant with the water bulk. Vasconcelo et al. [15] reported kf values ranging from 0.1 to 1.5 m d^− 1^. The average value of kf, obtained in this study is 2.79 m d^− 1^, which is higher than the aforementioned. In the new PVC pipeline of 110 mm diameter, there is no significant effect on the chlorine consumption due to the walls, this was because the pipe was new.

**Table 4:** Results obtained from K and kw for pipelines of different diameter and flow velocity

Diameter mm	Velocity (m s^− 1^)	kb (h^− 1^)	K (h^− 1^)	kf (m h^− 1^)	kw (m h^− 1^)	kw (m d^− 1^)
250	1.43	0.16	0.226	0.19	0.0046	0.11
200	1.09	0.16	0.222	0.15	0.0034	0.082
200	0.675	0.16	0.219	0.10	0.0033	0.080
160	1.51	0.16	0.271	0.21	0.0048	0.11
110	0.10	0.16	0.157	0.020	0.00004	0.0010
63	0.11	0.16	0.159	0.023	0.0001	0.0015

According to Table 4 it can be seen that that for a diameter of 200 mm and a velocity of 0.675 m s^− 1^, kw is 0.080 m d^− 1^ and for a pipeline with a smaller diameter of 160 mm and a velocity of 1.51 m s^− 1^, kw is 0.11 m d^− 1^, that is with a smaller diameter, kw effectively increases; meanwhile, at higher speed, kw increases. However, for a pipeline with a smaller diameter of 110 mm with a velocity of 0.10 m s^− 1^, kw is 0.0010 m d^− 1^; that is, in this case, kw decreases with decreasing diameters; meanwhile, at lower velocity, kw decreases. There is a positive correlation between velocity and kw; but there is no positive correlation between the diameter and kw. Therefore, these data confirm the abovementioned conclusion of Hallam that there is a positive correlation between the flow velocity and kw [17].

**Fig. 3:**
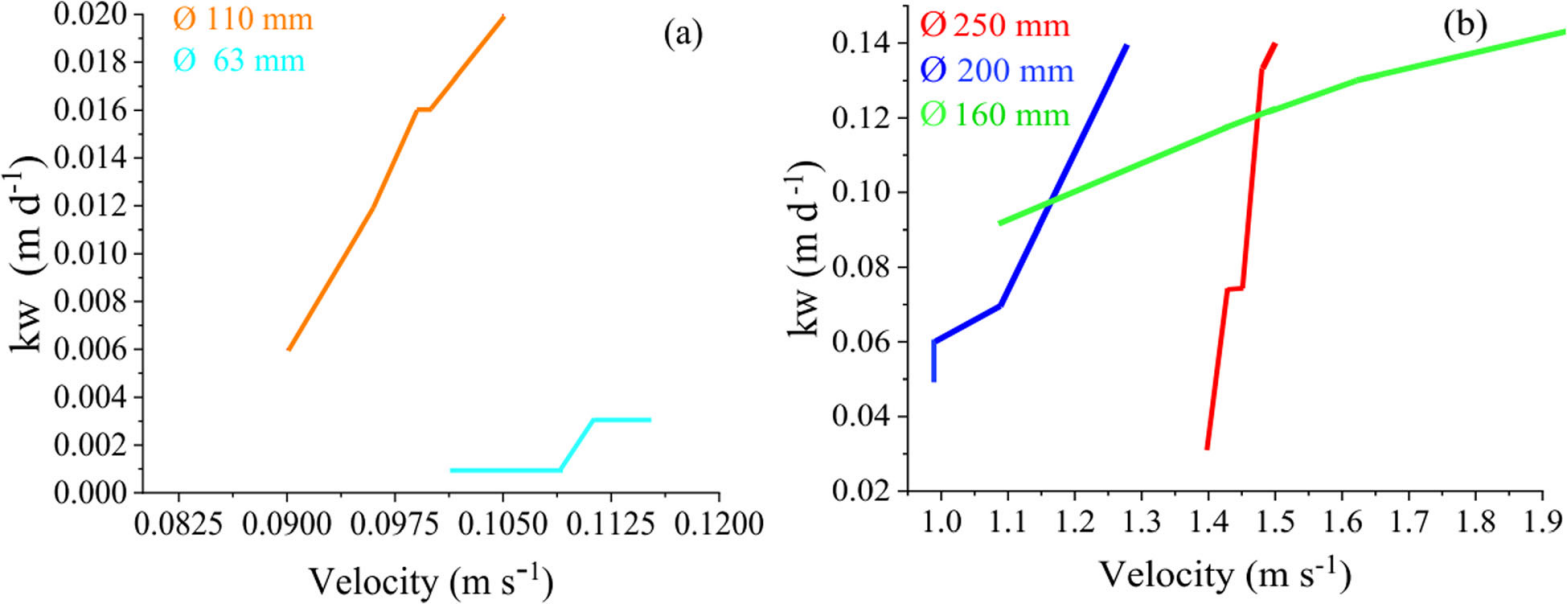
Variation of kw as a function of the velocity for the different diameters. **a** For diameter pipelines: 160, 200 and 250 mm **b** For diameter pipelines: 63 and 110 mm. These figures indicate that the chlorine decay rate with the wall is limited by the water flow velocity in the pipelines

In this study, kw values that varied between 0.0010 and 0.1141 m d^− 1^ were obtained. These values are within the range (0–0.15 m d^− 1^) reported in the EPANET Manual [29]. Figure 3 shows the wall decay constants as a function of flow rate for the different pipeline diameters. The curves for the 63, 110, 160 and 200 mm pipelines show similar trends of an increase over a wide velocity range. For the largest diameter of 250 mm, this increase is observed in only a small range of velocities. This indicates that the chlorine decay rate with the wall is limited by the water flow velocity in the pipelines.

**Fig. 4:**
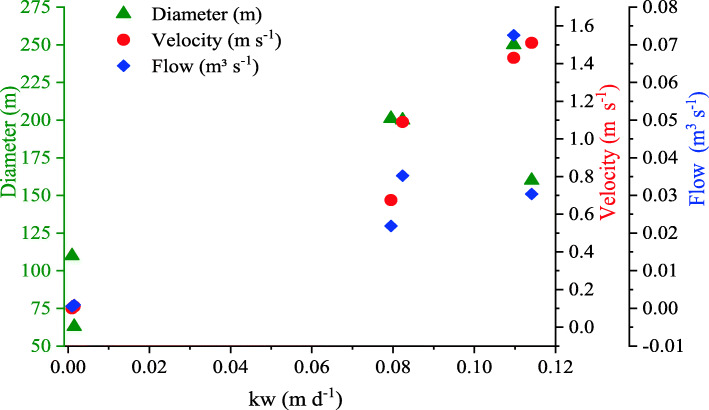
Variation of kw as a function of the pipe diameter, speed and water flow. In the figure, it can be seen that kw maintains a direct relationship with velocity, but not with diameter and flow

Due to the variations in pipeline diameters, flow rates in the distribution networks vary widely in space and time. Such variations are not taken into account when a single reaction constant is used to describe the decrease in the residual chlorine throughout the network. In this study, experimental evidence for a significant correlation between the pipe flow velocity and reaction rates with the wall of PVC pipelines was obtained (Fig. 4). It was also shown that as the velocity increases, the chlorine decay is more pronounced due to reaction with the pipeline walls.

### Chlorine decay model calibration and validation

**Fig. 5:**
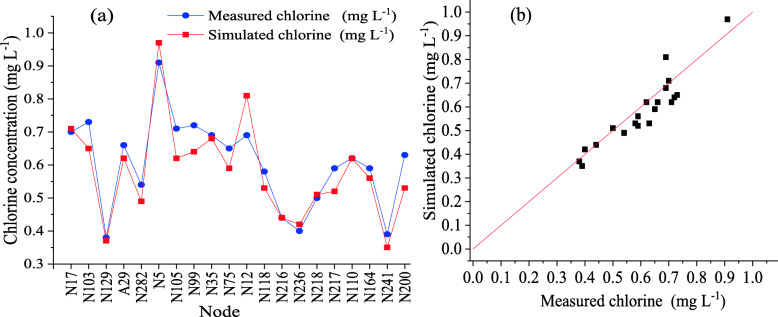
**a** Comparison of computed and measured chlorine concentration during the calibration **b** Pearson correlation results between computed and measured chlorine concentration during the calibration

EPANET 2.0 was used to model residual chlorine concentrations in the investigated area. The chlorine decay model was calibrated by the trial and error procedure after adjusting the values of kb and/or kw in several pipes. According to the general performance rating for the statistics recommended by Moriasi et al. [38], it was found from the results of the calibration efficiency that the Nash-Sutcliffe index (E) was 0.805, the RSR was 0.442, representing a “very good” rating, while the R was 0.9301, representing a significant positive rating. Therefore, it was possible to affirm that the model was adequately calibrated. The comparison of the observed and simulated chlorine concentrations in 20 nodes is presented in Fig. 5a. It is observed from this figure that the values measured in the field are close to the values simulated by EPANET. Therefore, it is concluded that the model was properly calibrated for the residual chlorine. Figure 5b shows the correspondence between the simulated values and those measured in the field with a Pearson correlation of 0.931.

**Fig. 6:**
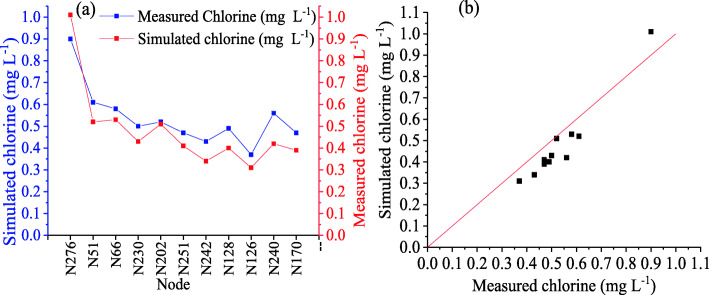
**a** Comparison of computed and measured chlorine concentration during the validation **b** Pearson correlation results between computed and measured chlorine concentration during the validation

The model was validated with 11 other data measured in the field that were different from those used in the calibration. The results are presented in Fig. 6a, and it is observed that the values measured in the field are close to the values simulated by EPANET. An E-index value of 0.769 was obtained, with the RSR index of 0.480 and R of 0.9898 (Fig. 6b), confirming that the chlorine decay model is valid, representing a good approximation for the actual water supply network.

It should be emphasized that the constant kb was determined experimentally. The constant kw was calculated once the constant K was determined in the field. After being entered into EPANET, the obtained values of kw and kb, it was possible to obtain real results in the modeling, according to the conditions of the DWDN under study. This strengthens the conclusions of the present study.

### Sensitivity analysis of the chlorine decay model

**Fig. 7:**
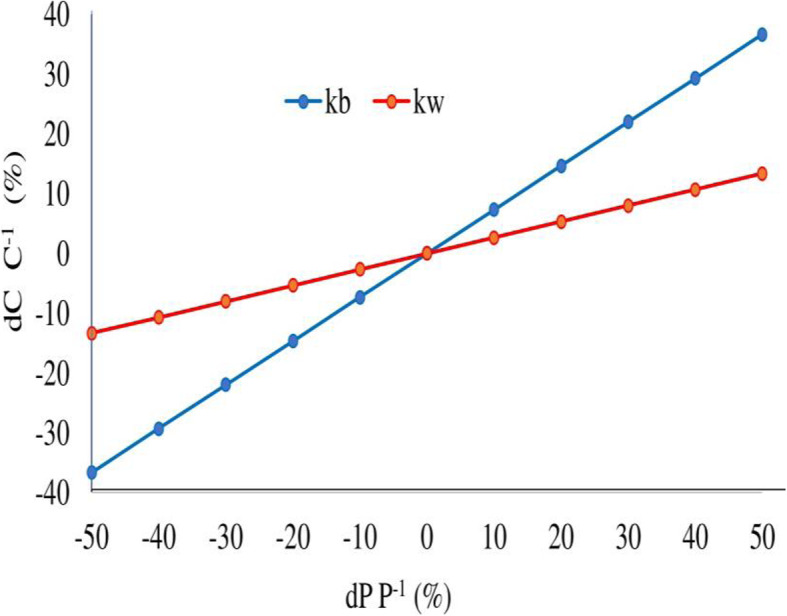
Sensitivity of the chlorine concentration simulated by the model through the changes in the kb and kw parameters in the distribution network. dC C^− 1^ is the relative change in the simulated chlorine concentration and dP P^− 1^ is the relative change in the values of the model parameters

Figure 7 shows the effects of the kb and kw parameters on the concentration of the residual chlorine in the distribution network. The kb parameter has the most significant effect on the concentration of the residual chlorine. A variation of 30% in kb decreases the chlorine concentration by 21%. The kw parameter shows the smallest variation effect on the concentration of simulated chlorine in the distribution network. A variation of 30% in kw decreases the chlorine concentration by only 8%. According to the sensitivity analysis, the factor that contributes the most to the residual chlorine decay is kb, while the residual chlorine decay is less sensitive to kw.

### Simulation. Temporal variation in residual chlorine concentrations

**Fig. 8:**
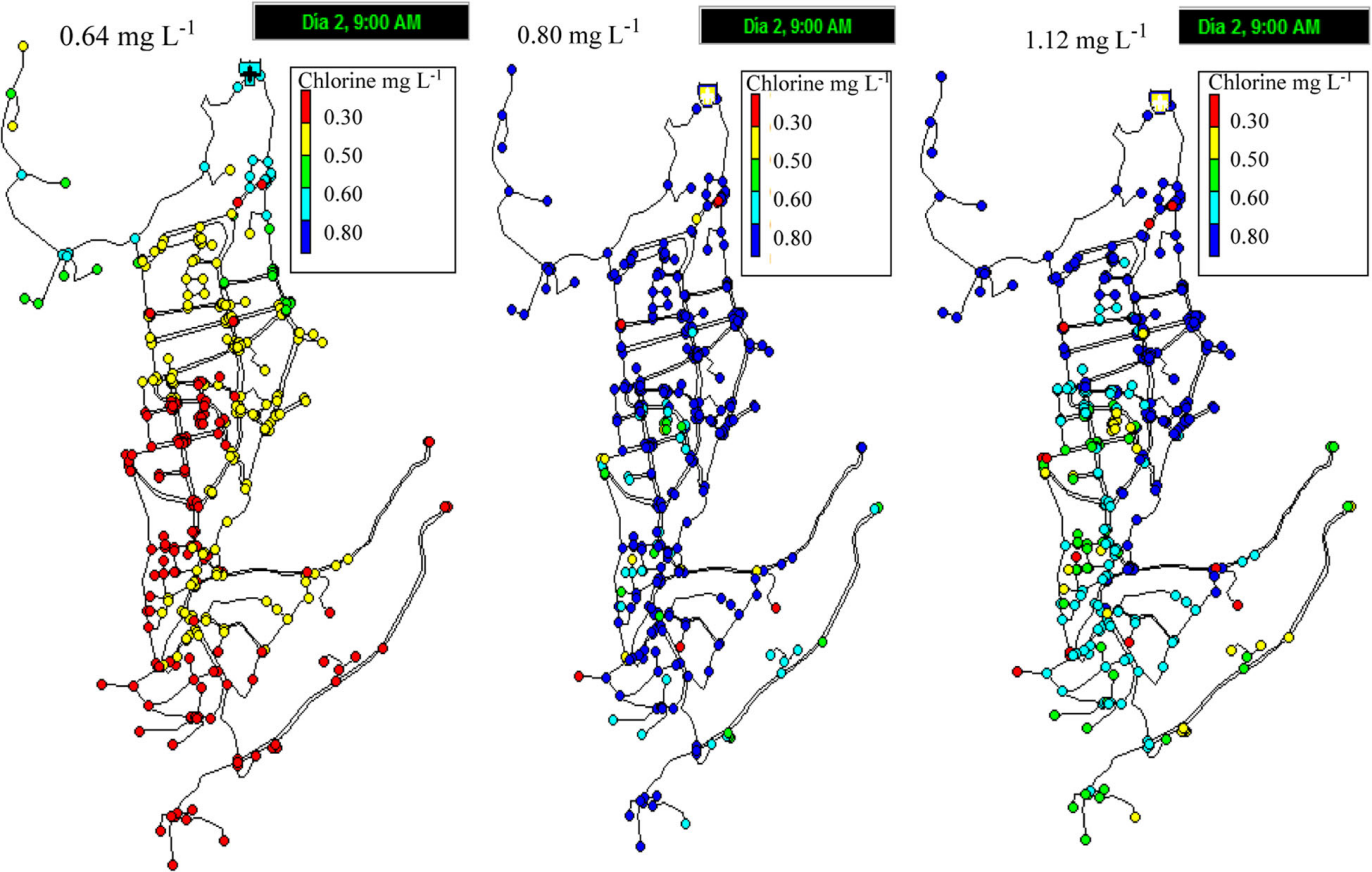
Simulated residual chlorine concentrations in the distribution network. **a** When the chlorine initial concentration in the distribution tank was 0.64 mg L^− 1^, **b** When the chlorine initial concentration was 0.80 mg L^− 1^, **c** When the chlorine initial concentration was 0.87 mg L^− 1^

**Fig. 9:**
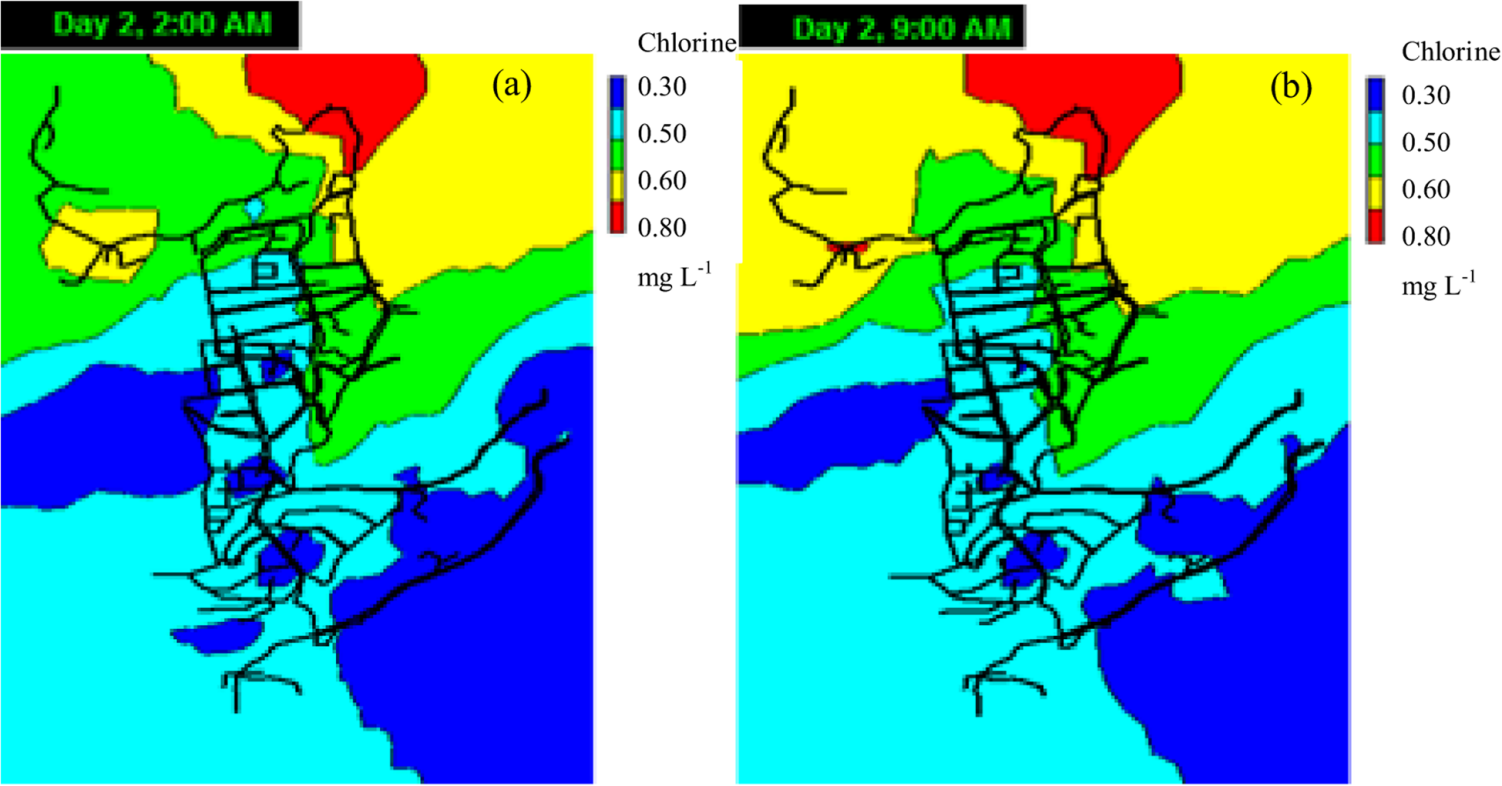
Simulation of residual chlorine concentration, using an initial concentration of 0.8 mg L^− 1^, which is the average value measured in the distribution tank. **a** At 2 am, residual chlorine concentrations in the 4% of the nodes are below 0.3 mg L^− 1^. **b** At 9 am, chlorine concentrations in 2.5% of the nodes are below regulation (0.3 mg L^− 1^)

The obtained chlorine decay model predicts the concentration of chlorine throughout the network. The residual chlorine concentration at the outlet of the distribution tank was in the range of 0.64–0.87 mg L^− 1^, with an average value of 0.80 mg L^− 1^. Independent simulations were performed using the lowest chlorine value measured in the tank (0.64 mg L^− 1^), the average value (0.80 mg L^− 1^) and the maximum value obtained in the tank (0.87 mg L^− 1^) (Fig. 8). It is observed that the initial chlorine concentration of 0.87 mg L^− 1^ is ideal for maintaining a residual concentration close to 0.3 mg L^− 1^ throughout the network, complying with the Ecuadorian standard. Conversely, an initial chlorine concentration of 0.64 mg L^− 1^ causes the chlorine concentration of 48% of the nodes to be below 0.3 mg L^− 1^. Using an initial concentration of 0.8 mg L^− 1^, which is the average value measured in the distribution tank, leads to the results that at the time of maximum consumption (9 am), chlorine concentrations of 2.5% of the nodes are below the regulation-specified value (10 out of 387 nodes). At the time of lowest consumption (2 am), concentrations of 96% of the nodes are above 0.3 mg L^− 1^, while 4% of the nodes do not comply with the regulation Ecuadorian (Fig. 9).

This result may be because water flow rates are reduced during the hours of lower consumption, increasing the chlorine decay constant and accelerating the decay of the disinfectant. The nodes that do not comply with the minimum concentration are located in the final parts of the branched network and are not necessarily the furthest away from the distribution tank; it is possible that there is an accumulation of the sediment in these network terminals that decreases the chlorine concentration. To mitigate the low chlorine concentrations for these few nodes, it may be possible to perform network washes in the nodes with low chlorine levels because these nodes are present in the terminals of the network.

Considering that the chlorine dose did not remain constant in the distribution tank during the day, it was observed that the chlorine measured in the distribution tank is considerably reduced at approximately 62.5% in the network. The chlorine in the distribution tank was 4 to 5 times higher compared to the points farthest from the network. Due to the high value of kb compared to other studies applied in various location, it is possible that the drinking water in this study contains a high concentration of dissolved organic carbon. Due to the rapid residual chlorine decay, it is suggested that the dissolved organic carbon content and the possible existence of trihalomethanes should be determined, as well as the NH_4_^+^ content [52].

### Use of the proposed model for epidemiological control

**Fig. 10:**
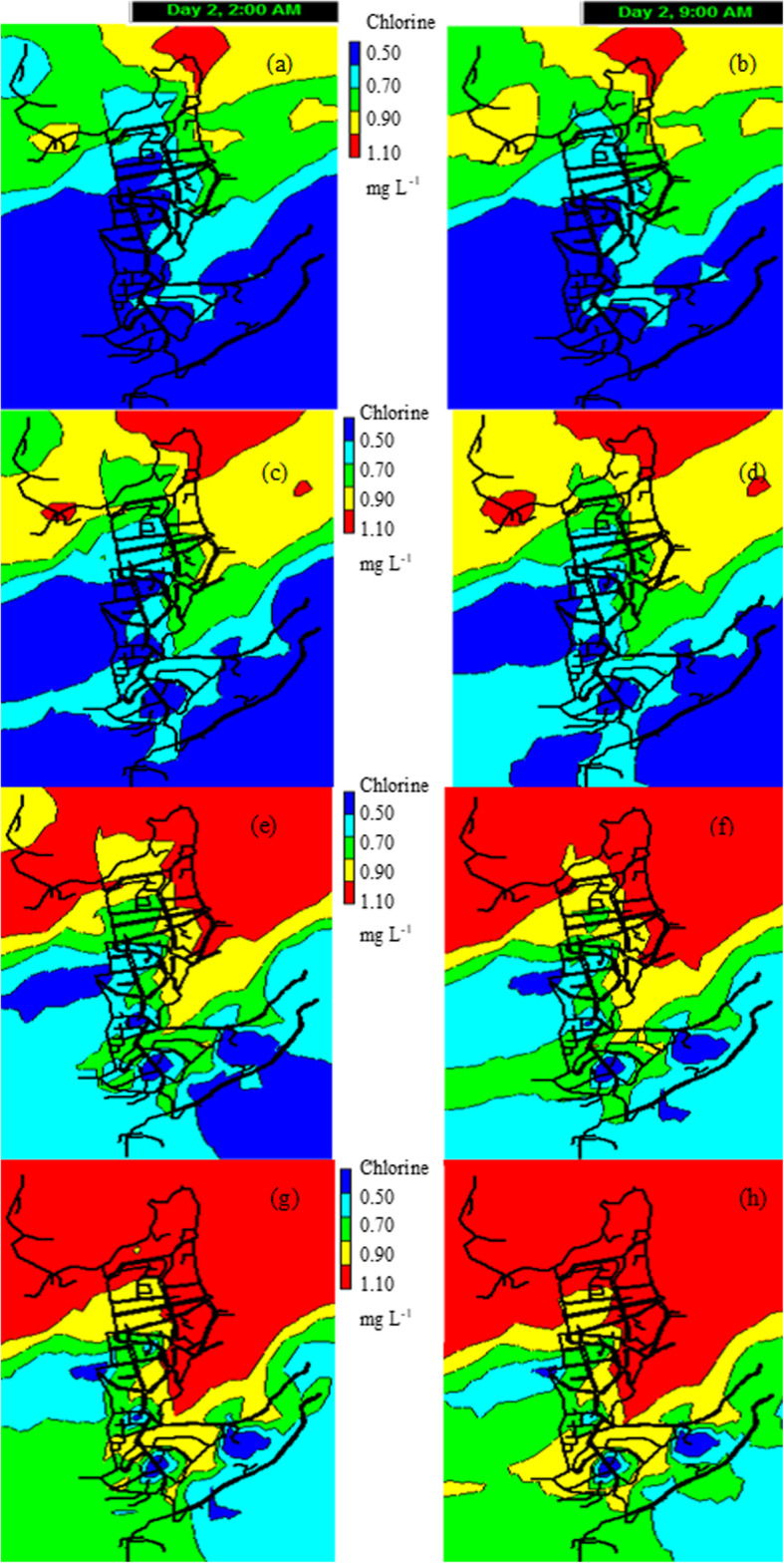
Simulation results, using the proposed model for epidemiological control. Compliance analysis according to WHO, residual chlorine > 0.5 mg L^− 1^. **a** When Co = 0.87 mg L^− 1^ in the distribution tank (DT), 2 am. **b** When Co = 0.87 mg L^− 1^ in DT, 9 am. **c** When Co = 1.2 mg L^− 1^ in DT, 2 am. **d** When Co = 1.2 mg L^− 1^ in DT, 9 am. **e** When Co = 1.5 mg L^− 1^ in DT, 2 am. **f** When Co = 1.5 mg L^− 1^ in DT, 9 am. **g** When Co = 2.0 mg L^− 1^ in DT, 2 am. **h** When Co = 2.0 mg L^− 1^ in DT, 9 am

To ensure health goals of chlorination in the current COVID-19 pandemic, WHO recommends that there should be a free chlorine dose concentration ≥ 0.5 mg L^− 1^ [5]. To analyze whether the WHO recommended in the DWDN under study is being met, a simulation was made using the highest value of chlorine measured in the tank (0.87 mg L^− 1^), but now, considering a value of 0.5 mg L^− 1^ as a minimum limit of residual chlorine. In Fig. 10a and b, it can be seen that a chlorine concentration of 0.87 mg L^− 1^ in the distribution tank is not enough to maintain a residual of 0.5 mg L^− 1^ throughout the network. The 45.2% of the nodes, at the time of minimum consumption (2 am) have chlorine levels below that recommended by the WHO for the current pandemic and 37.7% of the nodes were also below 0.5 mg L^− 1^ at the time of higher consumption (9 am). If we compare with the previous simulations, it can be seen that maintaining a concentration of 0.87 mg L^− 1^ in the distribution tank is enough to maintain levels greater than 0.3 mg L^− 1^ in practically all the DWDN, which is required by the Ecuadorian standard. Meanwhile, that same level of chlorine in the distribution tank would not allow minimum chlorine levels of 0.5 mg L^− 1^ in the DWDN.

Other simulations were made to establish the level of residual chlorine that would be necessary to maintain in the distribution tank, in order to find at least 0.5 mg L^− 1^ of the residual disinfectant in the DWDN. For which, levels of 1.2, 1.5 and 2.0 mg L^− 1^ of residual chlorine in the distribution tank were considered. The results of these simulations can be seen in Fig. 10. For each simulation, the percentage of nodes that had residual chlorine levels less than 0.5 mg L^− 1^ was calculated (Table 5).

**Table 5:** Percentage of nodes with chlorine concentration < 0.5 mg L^− 1^ after simulating different concentrations (0.87, 1.2, 1.5 and 2 mg L^− 1^) in the distribution tank

Chlorine concentration in the distribution tank (mg L^− 1^)	Node percentage (2 am) < 0.5 (mg L^− 1^)	Node percentage (9 am) < 0.5 (mg L^− 1^)
0.87	45.2	37.7
1.2	25.2	18.5
1.5	12.1	8.2
2.0	7.7	2.3

If the residual chlorine concentration in the tank were 1.2 mg L^− 1^, there would be 25.2% of nodes at 2 am (less water consumption) that would not comply with what is established by the WHO to combat the COVID-19 pandemic and 18.5% of the nodes would not comply at 9 am either (higher water consumption).

Meanwhile, by maintaining a residual chlorine concentration in the tank of 1.5 mg L^− 1^, there would be 12.1% of nodes at 2 am with chlorine levels less than 0.5 mg L^− 1^ and 8.2% at 9 am that would not comply with what is recommended by WHO.

Meanwhile, if the residual chlorine concentration in the tank were 2.0 mg L^− 1^, there would be 7.7% of nodes at 2 am that would not comply with the WHO established to combat the current pandemic and 2.3% of nodes that would not comply either at 9 am.

From Fig. 10a-d it can be observed that maintaining residual chlorine concentrations of 0.87 or 1.2 mg L^− 1^ in the distribution tank; at 2 am, more than 25% of the nodes (blue color) do not comply with what is established by the WHO. While at 9 am, more than 18% of the nodes (blue color) do not comply with what is established by the WHO. It is evident that by increasing the concentration of residual chlorine in the distribution tank, the percentage of nodes that do not comply with what is recommended by the WHO decreases.

From Fig. 10e to h it can be seen that maintaining residual chlorine concentrations of 1.5 or 2 mg L^− 1^ in the distribution tank; the percentage of nodes (blue color) that do not comply with what is established by the WHO drastically decreases, especially for levels of 2 mg L^− 1^. However, as can be seen in Fig. 10h, despite increasing the chlorine concentration in the distribution tank to 2 mg L^− 1^, there are still 2.3% of nodes with disinfectant levels lower than 0.5 mg L^− 1^. On the other hand, if these levels of 2 mg L^− 1^ were maintained in the distribution tank, there would be 8.9% of nodes with residual chlorine levels above 1.5 mg L^− 1^ (red color, Fig. 10g). When trying to comply with the WHO recommendation in all nodes, the nodes closest to the distribution tank would be affected by an increase in residual chlorine. As a result of high levels of residual chlorine, tastes and odors could be registered; as well as a potential problem of corrosion and formation of disinfection by-products, such as trihalomethane and haloacetic acids [10, 53]. As mentioned above, the nodes that do not meet the minimum concentration are located in the final parts of the branched network and are not necessarily the furthest from the distribution tank; in these nodes there may be accumulation of sediment, decreasing the concentration of chlorine. Therefore, it is recommended that the drinking water supply company implement a washing program in the identified terminal nodes.

An alternative to avoid high levels of residual chlorine in the distribution tank and therefore high dosages in the treatment plant, would be to implement booster stations in the distribution network. It is recommended as a potential research topic to analyze the possibility of implementing chlorine injection stations in certain nodes of the distribution network, the results of this research would allow the optimization of chlorination for the DWN, leading to considerable improvements in spatial uniformities and temporal concentrations of free chlorine.

We recommend that the proposed model be a support system to improve chlorine dosage in drinking water systems, considering the physical and hydraulic characteristics of the system, as well as the quality of the water supplied. For management purposes, the proposed model can help to make the right decision when it is necessary to increase residual chlorine levels in the DWDN, for example in cases of pandemic such as COVID-19 or cholera.

The results of the modeling made it possible to prediction the spatial and temporal changes of chlorine concentrations in the high zone network in Azogues city, Ecuador; considered the Ecuadorian norm and considering that recommended by the WHO for the current pandemic. It was also possible to determine the possible minimum levels of disinfectant to keep in the distribution tank to maintain a minimum of 0.3 mg L^− 1^ (Ecuadorian standard) and 0.5 mg L^− 1^ (recommended by the WHO for the current COVID-19 pandemic). To obtain better results in the simulation of residual chlorine, the decay coefficients of the disinfectant obtained by experimental tests in situ in the system under study should be used, since the decay coefficients should be determined with the conditions of the distribution network in study.

The implementation of programs to control adequate levels of residual chlorine in the distribution network are preventive and protective measures that should be considered to stop the spread of COVID-19.

## Conclusions

The focus of this research study was to develop a water quality model based on the residual chlorine decay. The implementation of this model made it possible to simulate the spatial and temporal changes of residual chlorine in the DWDN of the Azogues city, Ecuador, as a preventive and protective measure to combat the spread of COVID-19. The experimentally obtained values of kb (3.7 d^− 1^) and kw (0.066 m d^− 1^) were used to build the model in EPANET, which allowed to obtain modeling results more consistent with the reality of the DWDN. It is not advisable to use a single value of kw for all pipes of the network, since it has been shown that kw depends on the speed of the water, which is variable, because the pipes have different diameters and therefore there are different speeds for a same water flow.

To analyze the results of the simulation, the Ecuadorian standard and the one recommended by the WHO for the current pandemic were considered. The ideal residual chlorine values to be maintained in the distribution tank were simulated to ensure that the entire population has access to drinking water with adequate levels of residual chlorine. Considering the current concentration of residual chlorine in the distribution tank, it was evidenced that a level greater than 0.3 mg L^− 1^ is maintained in most of the nodes; meanwhile, there are 45.2% of nodes that maintain residual chlorine below what the WHO recommends in the current pandemic (0.5 mg L^− 1^).

The development of residual chlorine decay models in the DWDN enables potable water supply managers to have a useful tool for predicting the residual chlorine concentration throughout the network under a wide variety of changing hydraulic conditions. The implementation of these models will allow the selection of operational strategies and the optimization of chlorine disinfection practices in times of the SARS-CoV-2 pandemic.

## Data Availability

Not Applicable.
